# Impact of funded global health electives on career development of residents in a U.S. obstetrics and gynecology program: a cross-sectional survey

**DOI:** 10.1186/s12909-019-1536-8

**Published:** 2019-04-11

**Authors:** Suha Patel, Khady Diouf, Julianna Schantz-Dunn, Nawal M. Nour

**Affiliations:** 1 Department of Obstetrics, Gynecology, and Reproductive Biology, Brigham and Women’s Hospital, Boston, Massachusetts USA; 2 Harvard Medical School, Boston, Massachusetts 02115 USA

**Keywords:** Global health elective, Obstetrics gynecology residency

## Abstract

**Background:**

The aims of this study were to identify factors influencing participation in global health electives during residency and to understand the career impact of global health electives on alumni of an Obstetrics and Gynecology (OB/GYN) residency program.

**Methods:**

This was a cross-sectional, web-based survey of alumni of a residency program in the United States.

**Results:**

The response rate was 49%. Out of 73 respondents, 29 (39.7%) had completed a global health elective. Availability of funds and flexibility of elective time were the main enabling factors for participating in global health electives. Most participants of global health electives in residency reported a positive impact on their competence in domestic and global women’s health and on their career choices. Completing a global health elective in residency was associated with career work in global health and/or local health disparities (OR 4.62 (95% CI: 1.20–17.87)).

**Conclusion:**

Global health electives are important in the career development of residents. To foster OB/GYNs that continue global health and health disparities work, OB/GYN programs should give trainees the opportunity to participate in funded global health electives.

## Background

Over the last decade, global health electives have become increasingly common in U.S. residency training programs. Obstetrics and Gynecology has one of the lowest rates of global health residency training among the major specialties [1]. Yet, the role of obstetrician/gynecologists (OB/GYNs) in global health work is especially crucial given the importance of maternal mortality and perinatal mortality as major public health concerns worldwide.

In a recent survey of OB/GYN residents in the United States, resident motivation to pursue a global health elective was high. Over 80% said they would participate in a global health curriculum if it were offered and over 50% said they would use their vacation time to do so. However, barriers related to structure and budget of residency programs were identified as the most common reason this elective was not pursued [2]. In a larger survey of OB/GYN residents, over 95% rated global health experience in residency as somewhat important or very important [3]. Less than 20% of programs had structured options for global health work [3].

Our program started offering its residents funded opportunities for global health electives in 1997. In 2009, a global health division was established within the Department of Obstetrics and Gynecology at the institution. The goals of the division were to promote global health training and awareness among its residents and encourage cross institution partnership and exchange in research and clinical activities. We felt that alumni of a program with a structured setting for global health electives through mentorship and funding would be the perfect study group to determine which factors might influence their participation in global health experiences and whether provision of this training is worthwhile in their career development and other aspects of their professional development.

Given the delayed involvement of OB GYN residencies in the US in global health activities, few studies have assessed the feasibility and impact of global health electives on OB GYN resident training in the United States [2, 3]. To our knowledge, this is the first study to investigate these questions within a structured setting with explicit opportunities in global health electives. The primary aim of this study was to identify what factors influenced resident participation in global health electives. The secondary aim was to understand the impact of global health electives in residency on OB/GYN alumni of a large urban program over almost two decades.

## Methods

This study was a cross-sectional, anonymous survey of alumni at a large urban residency program who graduated between the years of 1997–2015. In February 2018, we emailed all 150 alumni who graduated during this period and for whom we had a correct email address and invited them to participate with a link to the online survey. After two weeks, non respondents received a reminder email. Enrollment was closed after a month. The survey was created and web-administered in REDCap [4] and responses were collected from February to March 2018. Respondents were included if they answered “yes” or “no” to the question “Did you have any global health experience during residency?”. There were 22 questions in the survey, ranging from demographic information to detailed questions about global health rotation site and activities. Most of the questions in our survey were taken with permission from a pediatric residency survey on global health experience that was previously internally validated [5]. Our survey was refined and piloted among faculty at our institution not involved with the study. This study was approved by the Partners Human Research Committee (2017P002551).

Results from the survey were analyzed using SAS version 9.4. Demographic characteristics including current occupation information were compared between alumni who did and did not participate in a global health elective. Chi squared test was used for comparison of categorical variables and t-test was used for comparison of mean age between groups. Fisher’s exact test was used for cell counts < 5. Multivariate logistic regression was performed to examine the association between participation in global health work during residency and current global health or health disparities work adjusted for age, multilingual status, specialty, and global health work experience prior to residency. Free text responses were analyzed using an inductive approach.

## Results

A total of 73 out of 150 (49%) eligible alumni participated in the survey. The average age of respondents was 41.4 years. Over 80% of survey respondents were female, reflecting the demographics of obstetrics and gynecology providers in the United States. Almost 40% of the respondents classified themselves as general OB/GYNs. All the major subspecialties were represented: Gynecologic Oncology, Maternal Fetal Medicine, Reproductive Endocrinology and Infertility, Urogynecology, Family Planning, and Minimally Invasive Gynecologic Surgery. Sixty-eight respondents (93.2%) reported being in clinical practice currently. 13.7% of respondents reported global health work and 9.6% reported health disparities work as part of their current careers. Most respondents spoke more than one language and participated in global health work before residency (Table 1). Twenty-nine respondents (39.7%) participated in global health electives during residency.

**Table 1:** Characteristics of survey respondents

	All Participants (*N* = 73)	Participants with global health experience in residency *N* = 29	Participants without global health experience in residency *N* = 44	*P* value
Graduation year				
1997–2001	15(20.5%)	4 (13.8%)	11 (25.0%)	0.69
2002–2006	18(24.7%)	8 (27.6%)	10 (22.7%)	
2007–2011	17(23.3%)	6 (20.7%)	11 (25.0%)	
2012–2015	20(27.4%)	9 (31.0%)	11 (25.0%)	
Unknown	3 (4.1%)	2 (6.9%)	1 (2.3%)	
Gender				
Female	61(83.6%)	24 (82.8%)	37 (84.1%)	1.00
Male	11(15.1%)	4 (13.8%)	7 (15.9%)	
Unknown	1 (1.4%)	1 (3.5%)		
Age of respondents				
Mean (std)	41.4(6.1)	40.3(5.7)	42.2(6.3)	0.20
Current specialty				
General OB/GYN	29 (39.7%)	14 (48.3%)	15 (34.1%)	0.33
Subspecialty	44 (60.3%)	15 (51.7%)	29 (65.9%)	
Marital status				
Married	56(76.7%)	19 (65.5%)	37 (84.1%)	0.29
Single	7(9.6%)	4 (13.8%)	3 (6.8%)	
Divorced/separated	3(4.1%)	2 (6.9%)	1 (2.3%)	
Serious relationship	5(6.8%)	3 (10.3%)	2 (4.6%)	
Unknown	2 (2.7%)	1 (3.5%)	1 (2.3%)	
Parent				
Yes	23(31.5%)	10 (34.5%)	13 (29.6%)	0.80
No	50(68.5%)	19 (65.5%)	31 (70.5%)	
Languages spoken				
English only	22(30.1%)	6 (20.7%)	16 (36.4%)	0.20
Multiple languages	50(68.5%)	22 (75.9%)	28 (63.6%)	
Unknown	1 (1.4%)	1 (3.5%)		
Global health experience before residency				
Yes	45(61.6%)	22 (75.9%)	23 (52.3%)	0.05
No	28(38.4%)	7 (24.1%)	21 (47.7%)	

Potential factors associated with completion of global health work in residency were examined. Respondents who reported global health experience before residency were more likely to participate in global health work during residency (Table 1).

**Fig. 1:**
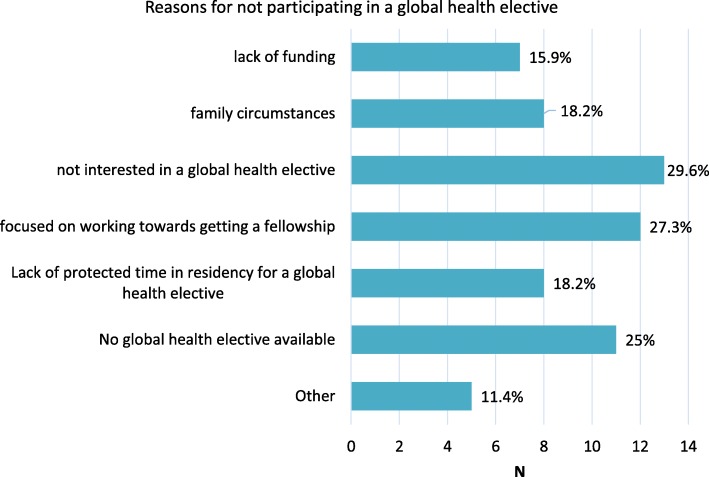
Reasons for not participating in a global health elective during residency (N = 44)

Among the 44 respondents who did not participate in global health electives during residency, the most common reasons for not participating were lack of interest (29.6%) and needing to focus on obtaining a fellowship (27.3%). These were followed closely by perceived lack of access to a global health rotation (25%). Lack of protected time, lack of funding, and family circumstances were also commonly reported barriers to participation (Fig. 1). Other reasons for not participating (written responses) were: security issues at planned elective site, opted not to pursue this work during residency.

**Fig. 2:**
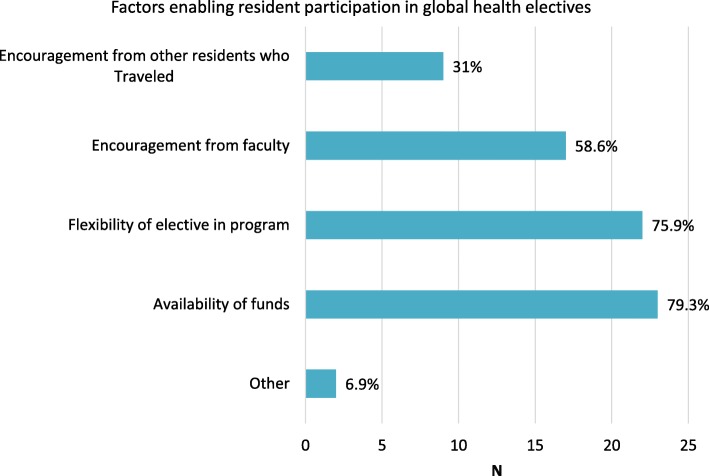
Factors enabling resident participation in global health electives during residency (*N* = 28). Percentage is out of the 29 respondents who did global health work in residency

The most common enabling factors for participation in global health work during residency were availability of funds and flexibility of elective time in the program, followed by encouragement from faculty (Fig. 2). Almost a third reported encouragement from other residents as enabling. In the free text, one respondent cited family connections as helpful and another respondent shared that taking his/her own initiative to find a project and a mentor at a different institution was an enabling factor.

Alumni who reported global health electives during residency were asked to report characteristics of their global health experiences. The median length of elective time for this experience was 5 weeks (IQR 4–6 weeks). 18 (62%) went to Sub-Saharan Africa for their global health electives. Other sites were in Central America, the Caribbean, South Asia, Europe, and the Middle East. 18 respondents (62%) went to urban global health sites and 5 went to rural sites (17.2%). 12 respondents (41.4%) reported returning to the same site or another site for global health work after their residency experience.

Alumni who undertook global health electives in residency reported several outputs from their experiences including improvements in their clinical skills, involvement in research and contributions to education (Table 2). Of the four respondents who reported other outputs, three respondents specified what these were in free text format. One reported improved surgical skills, one said that she/he provided expertise for a study, and another respondent gained perspective that is now useful in daily clinical practice working with immigrant and refugee women.

**Table 2:** Outputs of resident global health electives

	N (%)
Any output	24 (82.8%)
Personally improved clinical exam skills	13(44.8%)
Personally improved teaching skills	7(24.1%)
Personally improved research skills	6(20.7%)
Formally taught residents, medical students and/or staff at host site	9(31.0%)
Presented a global health case to other residents upon return	11(37.9%)
Presented work at a national or international conference	7(24.1%)
Published work in a peer reviewed journal	4(13.8%)
Completed curriculum or developed educational tools for on-site medical trainees	7(24.1%)
Completed curriculum or developed educational tools for community education	3(10.3%)
Other	4(13.8%)

**Fig. 3:**
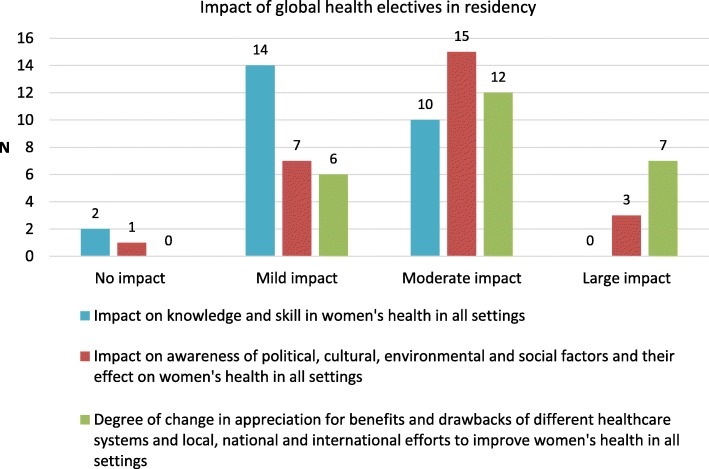
Impact of global health electives in residency

Respondents who completed global health electives in residency were then asked to assess how much their global health elective contributed to their knowledge and skill in women’s health in all settings (domestic and international). Most respondents reported a mild to moderate impact on knowledge and skills, and a mild to moderate impact on awareness of social, cultural, political and environmental factors affecting women’s health. Most of the respondents reported a moderate to large impact on their understanding of health systems and the delivery of women’s healthcare globally (Fig. 3).

Eighteen of the respondents with global health experience in residency (62.1%) were still doing global health and/or health disparities work in their current careers. Adjusting for global health experience before residency, age, multilingual status, and subspecialty status, alumni who did global health electives in residency were more likely to be involved with global health or health disparities work currently (OR 4.62 (95% CI: 1.20–17.87). 15 respondents (52%) reported that their global health experience in residency impacted their career development whereas 11 respondents did not think the experience had an impact on career. Respondents were asked to provide a description of how their global health elective affected their career, if at all. We received 12 responses. Three alumni shared how their experiences were career affirming or altering. Two alumni commented on the importance of having a global health elective in residency. Another two alumni shared that the experience gave them perspective on women’s healthcare delivery globally. Three alumni found the elective helpful in building their professional networks/collaborations to continue global health work. Two of the respondents mentioned cultural competence as a benefit of the experience. Three respondents wrote that their experience influenced their decision to pursue public health, policy, and/or research as part of their careers. One respondent wrote that the global health experience made him/her less optimistic about global health but influenced him/her to address health disparities in the United States.

## Discussion

We sought to understand enabling factors and barriers for global health elective participation and to describe the impact of global health electives on OB/GYN alumni from one residency program over almost two decades. The most important enabling factors for participants of global health electives were availability of funding and flexibility of elective time in residency. This is similar to findings from prior studies that identified funding and flexibility in time as common influencing factors for global elective participation cited by residents and program directors in the US [2, 6]. These barriers are surmountable and require conscious efforts and flexibility from programs to realize that these changes can affect the public health landscape in the long term by producing dedicated physicians willing to serve in areas of need. In our program for example, there was an overall increase in resident participation in global health electives, from 14% of respondents who graduated in 1997–2001 to 31% of respondents who graduated in 2012–2015. Increased participation was made possible by the gradual increase in support for these electives. First, internal funding for global health electives was implemented in 1997–1998 and additional funding mechanisms have become available over the years. Second, a departmental division in Global Obstetrics and Gynecology was created at the institution in 2009. The most common reasons that survey respondents did not participate in global health work during residency were working towards acceptance into subspecialty fellowship and/or lack of interest in global health. However, 25% of this group of alumni were not aware that a global health elective was an option during residency. Respondents who reported this barrier graduated between 1998 and 2012, a time during which some of their peers were participating in global health electives. Ensuring that all residents are aware of opportunities for global health experience during residency would have probably allowed a higher participation. Fifteen percent of those who did not participate in global health electives (7 respondents) perceived lack of funding as a barrier, yet all but one of these respondents graduated in the year 2000 or more recently (as recent at 2015) when funding should have been available to all residents. These reported barriers further support the need for improved publicity on opportunities to participate in global health electives during residency. Formalizing the global health elective in residency as an “opt-out” rotation would help reduce perceived barriers and increase participation. At least one institution in the US is having success with this approach [7]. Alternatively, a mandatory core curriculum in global health would provide opportunities for residents to be aware of issues in low resource settings and decide whether participation in an elective would be of interest.

Beneficial clinical, educational, and research outputs were commonly cited among alumni who did global health electives in residency. Most respondents felt that the experience contributed to their competence in women’s health both domestically and internationally. Over 50% of respondents who did global health electives in residency reported that the experience had a positive impact on their career choices. In some instances, respondents credited their global health experiences in their current work addressing health disparities in the U.S. Prior studies have also found that residents who train in underserved and international settings may be more likely to work in underserved areas in the US and abroad in the long term [6]. If a potential criticism of global health electives in residency is that areas of need in the US should be prioritized, results from this study and others emphasize that physicians with global health experience may be more likely to do future work in health disparities in local areas.

Our study has a few limitations. We cannot rule out the possibility of self-selection bias among the alumni who completed the survey. An internal department survey on global health participation was sent to recent alumni 5 months before our survey. Some of the younger alumni may have thought that both surveys were the same and ignored our survey request. This could have influenced the response rate to our survey. The authors of this paper were not involved in the internal department survey. The response rate for that survey was 44%.

Another possible limitation of this study is that the survey focused more on factors associated with participation in global health electives than on the content of available global health electives. Since the program did not have a unique site for the global health elective, residents may have had vastly different experiences owing to geography and culture (Asia vs Africa, urban vs rural) and activities during the elective (labor and delivery vs gynecologic surgery, research vs clinical work). A qualitative survey focusing on the survey respondents who participated in global health electives during residency would provide a more complete picture of the effect of global health electives during residency and allow us to identify room for growth and improvement in the electives accessible to the residents. We also did not investigate the possible impact of global health electives on host institutions since our study focused on impact among alumni who undertook global health electives. Prior studies have stipulated that global health electives may place a burden on host institutions and foster unequal partnerships, though most of these studies focused on medical students rather than specialty residents [8]. This will be worth investigating in future studies.

We found that global health electives during residency are important in the career development of trainees at our institution. We believe that these results are likely generalizable to U.S. OB/GYN trainees given the high interest in global health electives among national samples of residents [2, 3]. However, since a prior study showed that only 17% of US programs had structured electives similar to ours, our results may only be generalizable to these institutions [3]. Residency programs that are working to create structured global health electives for their residents may learn from our findings to remove barriers identified in our study as affecting participation in global health training during residency.

To decrease potential barriers, residency programs should offer flexible elective time, more formalized global health rotations, and access to funding. Since all residency programs may not have the capacity and resources to overcome these barriers, creation of a consortium of American OB/GYN residencies with global health partnerships could make supervised and structured rotations in multiple locations more accessible to U.S OB/GYN residents. In a national review of OB GYN programs in the United States with available global health training opportunities, only 17% of programs were identified as providing official electives [9]. Prior studies have cited global health training in residency as being beneficial to residents through strengthening their core competencies in an arguably more challenging setting, beneficial to faculty and institutions by providing opportunities for collaborative research and sharing of educational resources and beneficial for public health by training people who may be more likely to want deployment in underserved areas in the United States [6].

## Conclusions

This study shows that electives in global health are associated with residents’s future participation in careers focused on delivery of healthcare in areas with disparities. Allowing flexible and funded time during residency to pursue global health electives will indirectly add to the workforce available to resource-poor areas in the United States and abroad.
